# Frequency -Specific Air- Conduction and Bone - Conduction Outcomes after Stapedotomy

**DOI:** 10.22038/IJORL.2023.72213.3449

**Published:** 2023-09

**Authors:** Mohsen Rajati, Ali Ghanbari, Razieh Yousefi, Sadegh Jafarzadeh, Navid Nourizadeh, Mohammad Reza Sharifian, Imaneh Roshanzamir

**Affiliations:** 1 *Sinus and Surgical Endoscopic Research Center, Mashhad University of Medical Sciences, Mashhad, Iran. *; 2 *Student Research Committee, Mashhad University of Medical Sciences, Mashhad, Iran.*; 3 *Department of Audiology, School of paramedical sciences, Sinus and Surgical Endoscopic Research Center, Mashhad University of Medical Sciences, Mashhad, Iran.*

**Keywords:** Air conduction, Bone conduction, Frequency-specific, Hearing, Stapedotomy

## Abstract

**Introduction:**

The aim of this study was to investigate hearing outcome of stapes surgery, considering the post-operative air and bone conduction (AC&BC) changes, in a frequency specific approach.

**Materials and Methods:**

This was a retrospective cohort study. A total of 245 ears (231 patients), who underwent Stapedotomy at our tertiary referral center in a period of 5 years were enrolled in the study. Pure tone audiometry (PTA) was evaluated preoperatively and one month postoperatively. AC, BC, and Air-bone gap (ABG) were documented. Moreover, one-year post-op PTA was also recorded for more than a quarter of the cases.

**Results:**

Overall, significant improvements were observed in AC thresholds with a mean AC gain of 20.44±13.64 dB. At higher frequencies the results were poorer (AC gain of 27 dB at 250 Hz vs 7 dB at 8000 Hz). ABG significantly improved at all frequencies after one month. BC thresholds were typically better after surgery. However, there appears to be a worsening trend in BC thresholds at frequencies higher than 2000 Hz. In 68 patients with 1-year follow-up, BC thresholds were slightly worse (but not statistically significant) at most frequencies, in comparison to the one-month results.

**Conclusions:**

Stapes surgery significantly improves air and bone conduction hearing, particularly at lower frequencies. Nonetheless, there exists a potential for sensorineural hearing loss (SNHL) at high frequencies. However, the changes are insignificant and not within the speech frequencies. Therefore, patients are typically satisfied with the hearing outcome of the surgery.

## Introduction

Otosclerosis represents a unique disorder involving endochondral bone remodeling that specifically affects the otic capsule, potentially resulting in conductive, mixed, or even sensorineural hearing loss due to cochlear sclerosis (1,2.( The utilization of stapes surgery as the preferred therapeutic approach for otosclerosis dates back to the 1950s, demonstrating consistently favorable outcomes in a majority of cases (3,4,5). Some degree of bone conduction (BC) threshold improvement occurs after stapes surgeries (3,6-10).

This phenomenon can be attributed to multiple factors, including reduction in the extent of ossicular fixation, alteration in middle-ear biomechanics and cochlear fluid dynamics, reduction in the degree of conductive hearing loss and consequent refinement in BC threshold measurement, as postulated by the Carhart effect and the overclosure theory (10). 

Historical data on stapes surgeries have indicated that stapedotomy offers superior gains in high-frequency hearing and reduces the risk of sensorineural hearing loss (SNHL) (11), an infrequent complication affecting fewer than 1% of cases (1,12-14). High frequency SNHL, has been described as self-limited, resolving within a month. Several hypotheses have been advanced to explain this occurrence, including perilymph aspiration during surgery, mechanical trauma, and vestibular bleeding leading to serous labyrinthitis (1,10,13,15).

It is evident that the post-surgical auditory outcomes exhibit variability across different frequency ranges. Multiple investigations have been conducted on the hearing outcome following otosclerosis surgery so far. Nonetheless, while these studies have provided valuable insights, there exists a degree of inconsistency in their findings pertaining to postoperative changes in BC thresholds across various frequencies. To address this uncertainty, we have undertaken a frequency-specific survey approach, aimed at obtaining a comprehensive understanding of this intricate phenomenon.

## Materials and Methods

The retrospective cohort study was conducted on otosclerosis patients who underwent Stapedotomy over a five-year period (2015-2020) at our tertiary referral center. Eligible participants exhibited conductive or mixed hearing loss, diagnosed through clinical history, otoscopy, tuning fork tests, pure tone audiometry (PTA), and impedance findings. Exclusion criteria encompassed individuals under 18 years old, those who had undergone revision stapes surgery, patients with congenital middle ear anomalies, and those with specific risk factors for hearing loss, such as diabetes mellitus, chronic otitis media, rheumatologic disorders, temporal bone trauma, exposure to ototoxic agents, noise-induced hearing loss, and those who failed to complete their 1-month follow-up. The cases with intra-operative findings other than otosclerosis were also excluded from the study. Ethical approval was obtained from the local ethics committee (code: IR.MUMS.MEDICAL.REC.1398.883), and patients provided written informed consent before participation.

Small fenestration stapedotomy was performed in all patients via the transcanal approach. The prosthesis used was a Teflon Piston in all the cases. A lobular fat graft was used for sealing the Oval Window around the prosthesis shaft. Surgical details, including prosthesis length and diameter, footplate thickness, extent of Oval Window opening and other significant occurrences, were meticulously documented.

Preoperative and 1-month postoperative standard pure tone audiometry (PTA) was conducted for all participants, with additional one-year post-op PTA data available for a subset of cases. 

Audiometry was administered by qualified audiologists in a sound-treated room using a calibrated audiometer (Interacoustics AC 40). Air conduction (AC) and bone conduction (BC) thresholds were determined via TDH 49 supra-aural headphones and B-71 bone vibrator, respectively. Postoperative air-bone gap (ABG) was calculated by subtracting postoperative BC thresholds from postoperative AC thresholds, in line with the guidelines established by the Committee on Hearing and Equilibrium of the American Academy of Otolaryngology-Head and Neck Surgery (AAO-HNS) (16). Due to limitations the intensity of BC stimulus at 8000 Hz, a formula was devised to indirectly estimate the 8000 Hz BC threshold. Considering the relative consistency of ABG across different frequencies, 4000 Hz ABG is probably not much different from the real 8000 Hz ABG. Therefore, we calculated the 8000 Hz BC threshold by subtracting 4000 Hz ABG from the 8000 Hz AC threshold.

Data analysis was executed using SPSS software (version 16). Descriptive statistics were employed, including calculation of relative frequencies for categorical variables and mean±SD for continuous variables. The normality of continuous data was assessed with the Kolmogorov–Smirnov test, followed by paired t-tests and other descriptive measures to evaluate changes in target variables. Pearson correlation coefficients were also computed, and statistical significance was set at a p-value of less than 0.05.

## Results

A total of 245 ears (from 231 patients), comprising 82(%35) men and 149(65%) women, were included in the study. The average patient age was 42.41 years (±11.06), spanning 18 to 75 years.

Significant improvements in AC thresholds were observed across all frequencies after one month, reaching maximum at 250 Hz. Notably, the magnitude of AC improvement diminished with higher frequencies, as detailed in (Table 1).

**Table 1:** Comparison of pre-operative and post-operative Air Conduction (AC) thresholds at different frequencies

**Frequency (Hz)**	**Mean pre-op AC (dB)**	**Mean post-op AC (dB)**	**AC gain (dB)**	**p-value**
250	53.80±13.83	26.56±15.22	27.18±17.42	0.000
500	52.99±13.71	27.39±19.29	25.53±20.49	0.000
1000	52.08±14.92	26.43±15.64	25.68±16.18	0.000
2000	48.14±16.73	27.40±16.68	20.76±14.99	0.000
4000	50.68±19.86	35.55±20.44	15.05±16.67	0.000
8000	53.36±22.34	45.55±23.44	6.6±15.82	0.000
				

The mean AC gain was 20.44 dB (±13.64). ABG also exhibited noteworthy improvement across all frequencies one month postoperatively, as outlined in Table 2.

**Table 2:** Comparison of pre-operative and post-operative Air Bone Gap (ABG) at different frequencies

**Frequency (Hz)**	**Mean pre-op ABG (dB)**	**Mean post-op ABG (dB)**	**ABG closure (dB) **	**p-value**
250	42.97±12.51	18.75±12.18	24.22±16.55	0.000
500	38.07±12.48	16.83±16.24	21.23±19.78	0.000
1000	33.15±13.13	13.44±10.16	19.71±15.88	0.000
2000	18.80±11.58	9.92±6.71	8.88±12.29	0.000
4000	27.10±13.41	13.70±10.07	13.40±15.55	0.000
8000*	27.10±13.41	13.70±10.07	13.40±15.55	0.000


**Fig 1:**
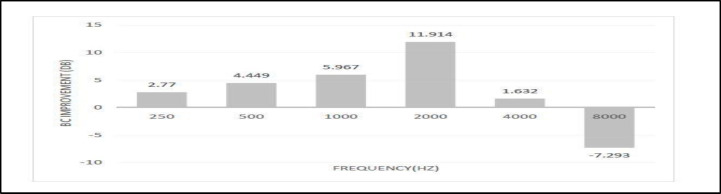
*Mean 1- month post-operative Bone conduction (BC)* *improvement at different frequencies* *(in 245 cases)*

BC thresholds displayed remarkable improvement within the 250-4000 Hz range, with the most substantial improvement (~12 dB) occurring at 2000 Hz, potentially attributable to the Carhart effect (Table 3). The trend in BC improvement exhibited an ascending pattern up to 2000 Hz, followed by a gradual downward slope beyond that point, with [calculated] BC thresholds deteriorating at 8000 Hz, as visualized in Figure-1.

The study did not record any cases of postoperative dead ear. However, a significant postoperative sensorineural hearing loss (SNHL) was observed in 4 cases (1.4%), characterized by BC deterioration of ≥15 dB at speech frequencies. No significant correlations emerged between hearing outcomes and age, patient gender, prosthesis length & diameter, or footplate thickness (p>0.05).

**Table 3:** Comparison of pre-operative and post-operative Bone Conduction (BC) thresholds at different frequencies

**Frequency(Hz)**	**Mean pre-op BC(dB)**	**Mean post-op BC (dB)**	**The difference (dB) **	**p-value**
250	10.87±7.62	8.05±6.74	2.77±8.30	0.000
500	15.12±9.60	10.65±8.61	4.45±9.06	0.000
1000	18.93±11.44	12.96±10.53	5.97±10.29	0.000
2000	29.49±13.95	17.47±13.71	11.91±13.03	0.000
4000	23.60±16.02	21.80±16.15	1.63±12.16	0.038
8000	26.59±19.78	32.22±20.98	-7.29±16.45	0.000

The same as 4 KHz, as explained in Methodology

Nonetheless, a noteworthy positive correlation surfaced between preoperative BC and BC improvement one-month post-surgery, as reflected by the correlation coefficient testing r (p-value), (Table 4). 

**Table 4:** Correlation between pre-operative Bone Conduction (BC) and BC improvement after one month

**Frequency (Hz)**	**250**	**500**	**1000**	**2000**	**4000**	**8000**
r (p-value)	0.639 (0.000)	0.572 (0.000)	0.535 (0.000)	0.485 (0.000)	0.368 (0.000)	0.284 (0.000)

This suggests that greater preoperative BC values corresponded to more substantial improvements in postoperative BC across all frequencies. However, the correlation was comparatively weaker at higher frequencies.

**Fig 2:**
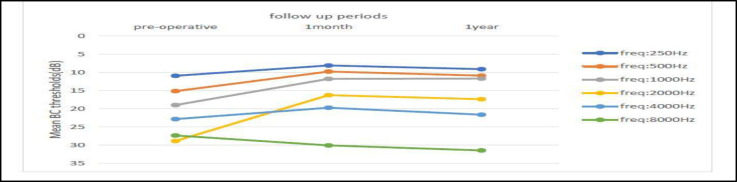
pre-operative, 1 month and 1 year post-operative mean Bone Conduction (BC) thresholds at different frequencies in 68 cases with 1-year follow-up

Around 25% of cases (68 out of 245) returned for their one-year follow-up. Analysis of this subset revealed slightly worsening in one-year post-operative BC thresholds across all frequencies, except at 1000 Hz, compared to one-month values (Figure 2). Nevertheless, these differences did not attain statistical significance. Furthermore, one-year post-operative AC and ABG values, across all frequencies, did not differ significantly from one-month values.

## Discussion

In designing our study, we aimed to investigate the possibility of persistant high-frequency sensorineural hearing loss (SNHL) following stapedotomy, challenging the prevailing notion of its transience. Our findings revealed noteworthy improvements in air conduction (AC) thresholds at all frequencies within one month after surgery, with the most remarkable gains noted at 250 Hz. However, the degree of hearing recovery (AC gain) was less substantial at higher frequencies, as evident by the lower AC gain at 8000 Hz compared to 250 Hz (Table 1). Interestingly, Bagger-Sjöbäck et al. reported hearing loss at ultra-high frequencies (above 10000 Hz) post-stapedotomy, which aligns with our observations (17).

Bone conduction (BC) thresholds exhibited improvements after surgery, particularly at 2000 Hz, attributed to the Carhart effect and the phenomenon of "overclosure"(10,18-21). This pattern of BC improvement was reversed above 2000 Hz, as seen in Figure-1. Post-operative SNHL was seen in 4 cases (1.4%). Vincent et al. reported a rate of SNHL of 0.5% on 3,050 stapedotomies (11). Among the subset of patients (68 out of 245) who completed the one-year follow-up, slight deterioration in BC thresholds was observed at most frequencies, although these differences were not statistically significant (Figure 2). This indicates that the initial decline in BC at 8000 Hz was not solely due to transient serous labyrinthitis, as suggested by some studies. Manuele et al. reported BC improvement at 250-4000 Hz after stapes surgery (21). Souza et al. also reported improvement in AC & BC thresholds consistent with that of Karimi et al., but the results were limited to 500, 1000, 2000 & 3000 Hz, and the higher frequencies were not reported (6,23). Moscillo et al. indicated similar results that were statistically significant only in younger patients (<45 years old) (3). Quaranta et al. reported the mean postoperative BC gain of 3.68 dB in partial stapedectomy and -0.02 dB in the stapedotomy group at the average 1000, 2000, and 4000 Hz frequencies (7). Aarnisalo et al. showed similar results after 6 months of follow-up but 13 dB deterioration was reported after 20 years follow up, probably caused by presbyacusis (8). Just et al. demonstrated a slight increase in the BC threshold in the frequency range between 4000 and 8000 Hz, 2–3 weeks after stapedotomy, which improves over time (15). Vincent et al. concluded that the mean four-frequency BC thresholds remained unchanged postoperatively, although worsened by 2.6 dB at 4000 Hz (11). The similar results were reported by Strömbäck et al., at 4000 Hz (12). Martin et al. found a deterioration of the BC thresholds at 4000 Hz and 8000 Hz by 6 dB and 8 dB, respectively, after stapes surgery, that improved at 9 months, and the final high-frequency loss was likely of little clinical significance (13). However, in our study the high-frequency BC did not improve over time. 

Our study uncovered a significant positive correlation between preoperative BC values and BC improvement one month post-surgery. Higher preoperative BC values were linked with greater postoperative BC improvements across all frequencies, similar to the findings of Sharaf et al. (24). Martin et al. revealed that an increased preoperative threshold was associated with a lesser chance of postoperative hearing loss, due to ceiling effects of more severe preoperative hearing loss (13).

In this study age, patient gender, prosthesis length & diameter, and footplate thickness did not significantly influence hearing outcomes, consistent with various previous reports. Martin et al., same as Strömbäck et al. concluded that age was not a predictor of postoperative high frequency hearing loss in the long term (12,13). Shah et al. found that age was not affecting the outcome of stapes surgery (25). Strömbäck et al. claimed better hearing results in obliterative otosclerosis.^11^ But Vincent et al. found correlation between obliterative otosclerosis and higher rate of postoperative SNHL (11). Faranesh et al. reported similar post-stapedotomy hearing results for the 0.4 and 0.6 mm prostheses with small advantage in BC gain and the overclosure parameter for the 0.6 mm prosthesis (26). While it has been suggested that stapedotomy yields significant improvements in high-frequency hearing (4000 and 8000 Hz) and low risk of immediate and delayed inner ear deafness (7,8), our findings raise questions about potential underlying mechanisms for high-frequency SNHL post-surgery. Adequate bone conduction is a fundamental prerequisite for a successful outcome of surgery (3). 

Possible contributors include damage to cochlear basal turn sensory cells during drilling and surgical manipulation, acoustic trauma from drilling-generated noise and resultant free radicals, and transient labyrinthine hydrops, anoxia, vascular sludging, bulging of the prosthesis into the vestibule, postoperative reparative granuloma, serous and suppurative labyrinthitis, endolymphatic hydrops and perilymph fistula. (1,10,12,13,15,17).

Some authors therefore recommended soft surgery using suctions with smaller luminal diameter and prophylactic medication with steroids and antioxidants and laser assisted stapedotomy (12,15).We believe that some other causes such as foreign body reaction to the prosthesis and altered mechanism of the mechanical energy transfer to the cochlea compared to the normal state (due to altered pattern of rocking motion around long and short axes of the foot plate at high frequencies and sectioning of stapedial muscle tendon) may also be involved (27).It's important to acknowledge limitations of our study, including the inability to directly measure BC thresholds at 8000 Hz using available clinical instruments. Our estimation of BC thresholds at 8000 Hz based on 4000 Hz ABG, may not be precise, but it serves to highlight a trend of downward sloping BC thresholds at higher frequencies.In limited previous studies on frequencies beyond 8000 Hz, mean threshold loss was observed at the frequencies of 10000–14000 Hz, despite the improvement of low-frequency hearing.^16^ Future investigations focusing on ultra-high frequencies may uncover a more comprehensive understanding of postoperative high-frequency SNHL, shedding light on occurrences beyond the scope of standard audiometry.

## Conclusion

Stapes surgery emerges as an effective intervention leading to substantial improvements in both air conduction (AC) and bone conduction (BC) hearing, particularly at lower frequencies. Nonetheless, there exists a potential for sensorineural hearing loss (SNHL) to manifest at high frequencies. Importantly, these changes, although present, do not achieve statistical significance and are not within the range of speech frequencies. As such, overall patient satisfaction with the hearing outcomes of the surgery remains high. However, our study highlights the need for continued investigation into potential adverse changes within the inner ear and basal turn of the cochlea, offering further exploration in future research endeavors.
